# Structural Elucidation and Moisturizing Potential of a Polysaccharide Derived from *Tremella mesenterica*

**DOI:** 10.3390/molecules31020278

**Published:** 2026-01-13

**Authors:** Geu-Rim Song, Hye-Ryung Park, Hye-Won Lee, Seo-Young Choi, You-Ah Kim, Byoung-Jun Park, Kwang-Soon Shin

**Affiliations:** 1 Development Team, Skin Research Team, Department of Skin & Natural Products Lab, Kolmar Korea Co., Ltd., Seoul 06800, Republic of Korea; thdrmfla12@kolmar.co.kr (G.-R.S.); hw0101@kolmar.co.kr (H.-W.L.); csy2696@kolmar.co.kr (S.-Y.C.); ahyou2@kolmar.co.kr (Y.-A.K.); a2001@kolmar.co.kr (B.-J.P.); 2 Department of Food Science and Biotechnology, Kyonggi University, Suwon 16227, Republic of Korea; pooh-lup@hanmail.net

**Keywords:** tremellastin, *Tremella mesenterica*, structural characterization, moisturization

## Abstract

*Tremella mesenterica*, commonly known as the yellow brain or golden jelly fungus, has been traditionally used for its medicinal properties. In this study, we elucidated the structural characteristics of *T. mesenterica* polysaccharide (TMP) and evaluated its potential moisturizing mechanism in vitro, comparing it to *Tremella fuciformis* polysaccharide (TFP) and hyaluronic acid (HA). TMP was isolated through enzyme assisted extraction and it has a molecular weight (MW) of approximately 143 kDa. We investigated the composition of mannose, xylose, glucuronic acid, and glucose as a ratio of 59.8 ± 0.3, 24.0 ± 1.2, 11.0 ± 0.8, 5.2 ± 0.0, respectively. Through methylation and GC-MS analysis, we discovered TMP was composed of a main chain of β-(1→3)-linked mannopyranoside, substituted with various side chains such as xylopyranoside, glucuronopyranoside, glucopyranoside at the C-2 or C-4 positions of the backbone. TMP upregulated the expression of key moisturizing-related factors compared to TFP and HA, such as aquaporin-3 (AQP3) with 55% and 57% at 25 and 50 μg/mL and hyaluronic acid synthase-2 (HAS2) with 22% at 25 μg/mL, as confirmed through qRT-PCR analysis. Additionally, TMP significantly enhanced the expression of filaggrin (FLG), a critical protein involved in skin barrier function, with 22% at 25 μg/mL. Immunocytochemistry (ICC) analysis further revealed that TMP achieved the highest improvement in hyaluronic acid synthase-3 (HAS3) protein levels by 475% at 50 μg/mL. While further in vivo studies are required to substantiate its functional moisturizing efficacy, these findings suggest that TMP serves as a promising moisturizing agent. The structural and functional properties of TMP provide a potential foundation for its application in diverse industries, including cosmetics, food, biopolymers, and pharmaceuticals.

## 1. Introduction

The genus *Tremella (family Tremellaceae)* is classified within the group of organisms commonly referred to as ‘jelly mushrooms’, characterized by their gelatinous fruiting bodies. The genus *Tremella* comprises over 120 species [1], many of which have been used in food and traditional medicine in Asian countries for centuries [1]. Among these, *T. fuciformis* is the best-known white jelly fungus. *T. fuciformis* has been extensively studied for its health benefits, which include enhanced immunity, lowered blood sugar, and antitumor and anti-aging effects [2]. Additionally, it is widely recognized for its moisture-preserving properties, which are attributed to its polysaccharides, which primarily consist of glucuronoxylomannan (GXM) [3].

*T. mesenterica*, commonly known as the golden jelly fungus, has been recognized for its various health benefits and medicinal applications. It exhibits a diverse range of therapeutic properties, including immunostimulation, anti-inflammatory, protection against radiation, hypocholesterolemic, hepatoprotective, anti-diabetic, and anti-allergic effects [4]. Tremellastin, a polysaccharide derived from *T. mesenterica*, is an acidic polysaccharide containing over 40–50% GXM [5]. Despite this potential, the structural properties and biological functions of *T. mesenterica* polysaccharide (TMP) have not been thoroughly investigated, whereas *T. fuciformis* polysaccharide (TFP) has been extensively studied for its biological activities [2,3].

Polysaccharides exhibit diverse physiological activities depending on their glycosidic linkages and structural features. More polar groups, such as hydroxyl and carboxylic groups, enable polysaccharides to trap and lock water molecules through hydrogen bonding, which contributes to their hygroscopic and moisturizing activity [6]. Additionally, polysaccharides have been shown to enhance the activity of human epidermal keratinocytes, promoting cell proliferation and stimulating the production of key skin moisturizing factors such as hydration proteins and hyaluronic acid [6,7]. They can also influence gene expression in deeper epidermal layers by interacting with cell surface receptors and activating signaling pathways, such as the MAPK pathway [8].

Through these mechanisms, polysaccharides contribute to improved skin hydration and overall moisturizing capacity [6].

The purpose of this study was to chemically elucidate TMP and evaluate its moisturizing potential by comparing it with that of TFP and hyaluronic acid (HA) in vitro. We aimed to highlight the potential applications of TMP and contribute to the development of advanced skin moisturizers.

## 2. Results and Discussion

### 2.1. Extraction of TMP

Polysaccharide extraction is a critical step for obtaining high-quality polysaccharides because it significantly influences their yield, chemical structure, and biological activity [2]. Among the various extraction methods, the most commonly used extraction method is hot-water extraction because of its simplicity and efficiency. However, this conventional method often results in a low yield and purity. To address these limitations, we conducted a multi-enzymatic extraction to enhance the yield and quality of the polysaccharides. We selected three specific enzymes (Viscozyme L, Pectinex XXL, and Performase GSM 80) based on their complementary functional roles in optimizing polysaccharide yield and purity. Viscozyme L is a multi-component carbohydrase, contains β-glucanase, pectinase, hemicellulase, and xylanase activities, which effectively hydrolyze β-1,3/1,6-glucan, a major component of the fungal cell walls, thereby facilitating polysaccharide release [9,10]. Pectinex XXL includes pectinase, hemicellulase, arabinanase, which indirectly weaken the cell wall and supplementarily enhance polysaccharide extraction [10]. Performase GSM 80, a protease derived from papain [11], degrades glycoproteins (e.g., mannoproteins) associated with the cell wall, thereby weakening the interactions between the cell wall and polysaccharides, removing protein impurities, and improving the purity of the extracted polysaccharides. The synergistic effect of combining Viscozyme L, Pectinex XXL, and Performase GSM 80 can be attributed to their complementary actions on the cell wall components. Viscozyme L and Pectinex XXL effectively hydrolyzed polysaccharides such as β-glucan and xylose-based polysaccharides, while Performase GSM 80 degraded proteins associated with the cell wall, facilitating further polysaccharide release.

This synergistic action contributed to a significant increase in the total polysaccharide content (TPC), which indirectly reflects the purity of the extracted polysaccharides, and polysaccharide yield, as summarized in Table 1. The enzymatic treatment process resulted in significantly improved both yield and TPC compared to the untreated control. The optimized enzyme ratio (Viscozyme L:Pectinex XXL:Performase GSM 80) was determined to be 1:1:2, resulting in a yield of 26.74 ± 2.94%, which is approximately four times greater than that of the untreated control (6.21 ± 1.61%). These findings indicate that multi-enzymatic treatment is superior to single-enzyme treatment and that GSM 80 serves essential functions in enhancing both the yield and purity of TMP when mixed with other enzymes.

Under the optimized conditions, the yield of TMP was approximately 11 times higher than that of TFP (2.28 ± 2.06%). Additionally, TPC of TMP was 98.82% compared to 56.66% for TFP. These results indicated that TMP contains more active components and may exhibit greater biological activity than TFP.

### 2.2. Structural Characterization Results of TMP

#### 2.2.1. MW and Monosaccharide Composition of TMP

To evaluate the chemical properties of the TMP, its MW and monosaccharide composition were determined. The MW of TMP, as determined via high-performance size-exclusion chromatography (HPSEC) using a Superdex 75 GL column, was estimated to be 143 kDa (Supplementary Figure S1). Monosaccharide analysis (Table 2) revealed four types of monosaccharides, with mannose (Man) (59.8%), xylose (Xyl) (24.0%), glucose (Glc) (5.2%), and glucuronic acid (GlcA) (11.0%) as the major constituents.

These results confirmed that the polysaccharide exhibited a monosaccharide composition similar to that previously reported for *T. mesenterica* [4]. In contrast, analysis of polysaccharides from *T. fuciformis* revealed a comparable composition; however, fucose was also detected [1]. These findings suggest that the monosaccharide composition may be influenced by the species of *Tremella*.

#### 2.2.2. Glycosidic Linkage of TMP

The glycosidic linkage pattern of TMP was investigated using methylation analysis to elucidate its primary structural features. As summarized in Table 2, TMP predominantly consists of 18 types of glycosyl linkages, including mannose, xylose, glucuronic acid, and glucose. For mannose residues, the major linkages included terminal Manp (11.8%), 3-linked Manp (19.6%), 2,3-linked Manp (30.1%), 3,4-linked Manp (2.0%), 2-linked Manp (0.9%), and 2,3,4-linked Manp (0.9%). Xylose residues were mainly present as terminal Xylp (4.7%), 3-linked Xylp (6.7%), and 2-linked Xylp (0.7%). In the case of glucose, the glycosidic linkages were terminal Glcp (1.0%), 2,3-linked Glcp (4.0%), 4-linked Glcp (0.6%), and 3-linked Glcp (0.5%). Glucuronic acid residues were primarily represented by terminal GlcAp (2.5%), 4-linked GlcAp (10.2%), 2-linked GlcAp (1.7%), 3-linked GlcAp (0.8%), and 2,4-linked GlcAp (1.1%).

**Figure 1:**
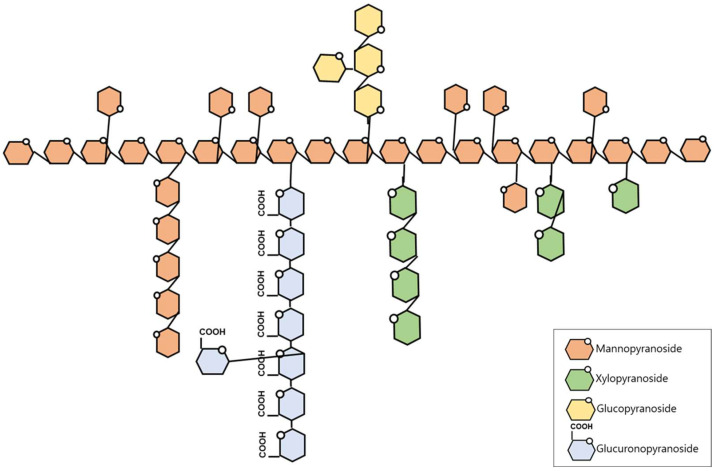
The proposed structure of TMP.

Based on methylation-derived linkage distributions, a tentative structural model of TMP is proposed in Figure 1. TMP appears to contain a mannan-rich backbone in which →3)-linked Manp residues are prevalent, with substitutions mainly at O-2 and, to a lesser extent, at O-4 as suggested by the presence of 2,3-linked and 3,4-linked Manp residues. The relatively high proportion of 3-linked Manp (19.6%) is consistent with the presence of β-(1→3)-linked mannosyl segments within the polysaccharide. In addition, the detection of 3-linked Xylp (6.7%) indicates the presence of →3)-linked xylosyl residues; however, the presence of these residues does not allow a definitive assignment of a linear β-(1→3)-xylan backbone.

TMP also shows linkages consistent with glucuronosyl- and glucosyl-containing side-chain components. Specifically, the dominance of 4-linked GlcAp (10.2%), together with terminal and substituted GlcAp residues, suggests the presence of →4)-linked glucuronosyl segments with branching points. Similarly, the presence of 3-linked Glcp and terminal/substituted Glcp residues suggests glucosyl segments with branching. Because methylation analysis alone cannot unambiguously define the exact sequence and length of these side chains, these features are presented as a schematic interpretation derived from linkage types and their relative abundances.

The previously reported TMP structure [12,13] was defined by a β-(1→3)-linked mannan backbone with side chains branching at the C-2 or C-4 positions. The side chains consist of 2-linked xylan, which occurs in the form of xylobiose or xylotriose, together with a single glucuronic acid residue branching from the main chain. Although the monosaccharide composition was identical to that of tremellastin, which was identified in the present study, the linkage profile of TMP suggests a higher degree of branching and structural heterogeneity than the previously reported *T. mesenterica* polysaccharide. Additionally, TMP exhibits distinct differences from TFP [12,14]. TFP possesses a relatively simple structure consisting of a 3-linked mannan backbone with single GlcAp, Fuc, and Manp residues branching at the C-2 position of the main chain, whereas only the Xylp residues extend in pairs. In contrast, TMP has a much more highly branched and complex structure, which may contribute to differences in biological activity.

### 2.3. Cytotoxicity Assessment

#### Cytotoxicity of TMP, TFP, HA

**Figure 2:**
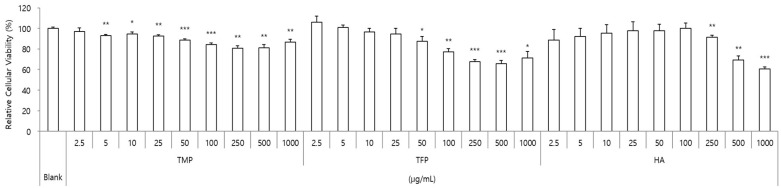
Cytotoxicity of TMP, TFP, and HA in HaCaT cells. HaCaT cells were exposed to various concentrations (2.5–1000 μg/mL) for 24 h, and cell viability was assessed. TMP showed no cytotoxicity, whereas TFP and HA reduced cell viability at concentrations above 100 μg/mL and 500 μg/mL, respectively. Based on these results, 10, 25, and 50 μg/mL were selected for further experiments. Data are presented as the mean ± SD from three independent experiments. * *p* < 0.05, ** *p* < 0.01, *** *p* < 0.001 as compared to the control group.

HaCaT cells were incubated with different concentrations (2.5–1000 μg/mL) of TMP, TFP, and HA for 24 h and the percentage of cell viability was evaluated. As presented in Figure 2, TMP exhibited no cytotoxicity across all tested concentrations. In contrast, TFP showed cytotoxic effects at concentrations above 100 μg/mL, and HA decreased cell viability at 500 μg/mL. Therefore, the maximum final concentrations for TMP, TFP, and HA were set at 50 μg/mL and 10, 25, 50 μg/mL concentrations were treated for subsequent experiments to compare their activities at equal concentrations.

### 2.4. Mechanisms of TMP in Skin Cell Moisturization

#### 2.4.1. Gene Expression Analysis of Moisturizing Related Markers

**Figure 3:**
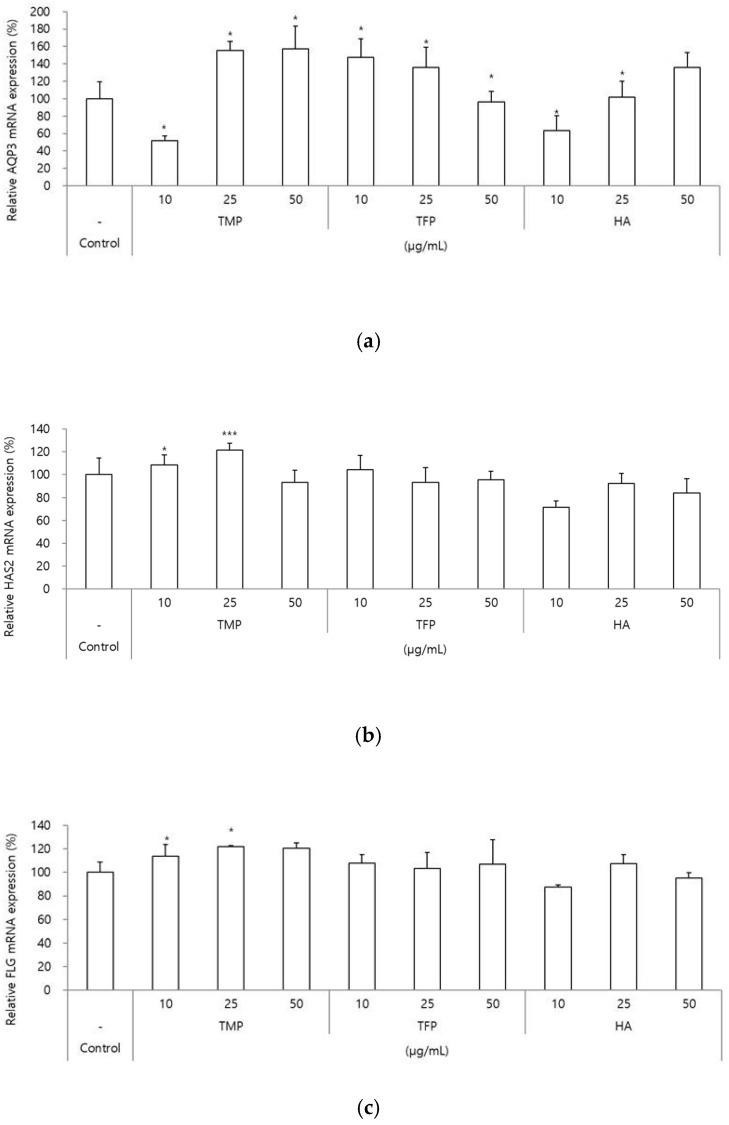
Gene expression analysis of moisturizing and barrier-related markers (*AQP3, HAS2*, and *FLG*) in HaCaT cells treated with TMP, TFP, and HA. (a–c) HaCaT cells were treated with TMP, TFP, and HA at different concentrations (10, 25, and 50 μg/mL), and the expression levels of *AQP3, HAS2*, and *FLG* were analyzed. TMP showed superior effects on the expression of moisturizing and barrier-related genes compared to TFP and HA. Data are presented as the mean ± SD from three independent experiments. * *p* < 0.05, *** *p* < 0.001 as compared to the control group.

The expression levels of genes related to moisturizing and barrier functions (AQP3, HAS2, and FLG) were evaluated after treatment with TMP, TFP, and HA. One of the moisturizing-related markers, AQP3, which is predominantly expressed in keratinocytes and involved in water and glycerol transport [15], resulted in significant increases in gene expression of 55% and 57% when exposed to TMP at 25 and 50 μg/mL, respectively. In contrast, TFP showed moderate effects, with 47% and 36% increases at 10 and 25 μg/mL, respectively, whereas HA demonstrated a 36% increase at 50 μg/mL (Figure 3a).

For HAS2, mRNA level, TMP treatment at 25 μg/mL led to a 22% increase in gene expression, whereas no significant effects were observed for TFP or HA treatment (Figure 3b). HAS2 is one of three isoforms of hyaluronan synthases (HAS1, HAS2, and HAS3), which are membrane-bound enzymes and the most abundantly expressed isoform in keratinocytes [16].

FLG, which is a structural protein that is fundamental in the development and maintenance of the skin barrier [17], resulted in increased gene expression with 22% and 20% at 25 and 50 μg/mL, respectively. Significant effects were not detected for the TFP and HA treatments (Figure 3c).

These results suggest that TMP may interact with skin hydration and barrier-related mechanisms more effectively than TFP and HA in vitro. The higher expression levels of HAS, AQP3, and FLG observed with TMP are likely attributed to its structural characteristics. While hyaluronan (HA) is a linear polyanion with a repeating disaccharide structure [(1→3)-β-dGlcNAc-(1→4)-β-d-GlcA] [18], TMP exhibits a highly branched structure composed of glucuronic acid, mannose, xylose, and glucose. This branched structure increases the surface area for hydrophilic functional groups to interact with water molecules, potentially enhancing moisture retention. Additionally, the lower TPC content in TFP compared to TMP suggests fewer hydrophilic functional groups available for water molecule interaction, which may explain the relatively lower expression levels of moisture-related markers observed with TFP.

#### 2.4.2. ICC of HA Synthesis Induced by TMP

**Figure 4:**
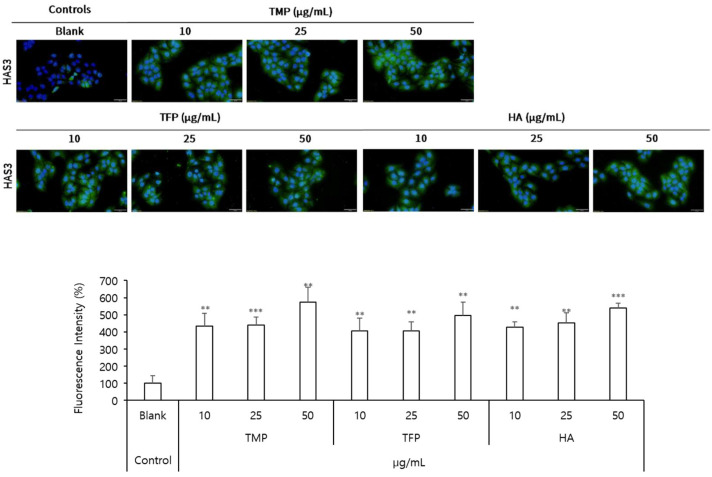
Immunofluorescence analysis of HAS3 protein levels in HaCaT cells incubated with TMP, TFP, and HA. HAS3 protein levels were analyzed to evaluate the moisturizing effects of TMP, TFP, and HA. TMP showed the highest increase in HAS3 protein expression among the tested samples, highlighting its superior efficacy in enhancing skin hydration. Green indicates HAS3 expression (Scale bar = 50 μm). Data are presented as the mean ± SD from three independent experiments. ** *p* < 0.01, *** *p* < 0.001 as compared to the control group.

To further evaluate the moisturizing effects of TMP, HAS3 protein levels were analyzed using immunofluorescence imaging (Figure 4). At a concentration of 50 μg/mL, TMP significantly increased HAS3 protein levels by 475%, demonstrating the highest effect among the tested samples. In comparison, TFP at 50 μg/mL increased HAS3 protein levels by 397%, whereas HA at the same concentration induced a 439% increase. These findings suggested that TMP exhibited superior efficacy in enhancing HAS3 protein expression, further supporting its potential as an effective agent for improving skin hydration.

## 3. Materials and Methods

### 3.1. Materials and Reagents

*T. mesenterica* was obtained from Korea Pyogo Farm (Hoengseong, Republic of Korea), and the enzymes (Viscozyme L, Pectinex XXL, and Performase GSM 80) were purchased from Daejongzymes Co. (Seoul, Republic of Korea). For determination of TPC, 98% sulfuric acid (Daejung, Siheung, Republic of Korea), 98% phenol Duksan (Seoul, Republic of Korea), and glucose (Sigma-Aldrich Co., St. Louis, MO, USA) were used.

For the analysis of structural features, trifluoroacetic acid (TFA), 1-phenyl-3-methyl-5-pyrazolone (PMP), methyl iodide (CH_3_I), sodium borohydride (NaBH_4_), mannose, xylose, glucose, and glucuronic acid were acquired from Sigma-Aldrich Co. (St. Louis, MO, USA). Unless otherwise specified, all chemicals utilized in this study were of analytical grade.

The HaCaT human keratinocyte cell line was supplied from the Kyung Hee University (Seoul, Republic of Korea). The cells were cultured in Iscove’s Modified Dulbecco’s medium (IMDM; 12440-053) containing 10% fetal bovine serum (FBS; 16000-044), 1% antibiotics and antimycotics (15240-062), 0.25% Trypsin-EDTA (25200-056), and phosphate-buffered saline 7.4 (PBS; 10010-023) provided by Gibco (Grand Island, NY, USA). HA (200–400 kDa) was obtained from Bloomage Biotech Co. (Jinan, China). Cell cytotoxicity was measured using Thiazolyl Blue Tetrazolium Bromide (MTT assay; M2128) delivered from Sigma-Aldrich, dimethyl sulfoxide (DMSO; 1380), and Duksan Corp. (Ansan, Republic of Korea). For the determination of Aquaporin 3 (AQP3), HA synthase 2 (HAS2), and Filaggrin (FLG) mRNA levels, Trizol (1559601), nuclease-free water (AM9932) purchased from Invitrogen (Carlsbad, CA, USA), cDNA Synthesis kit (ET21100) purchased from PhileKorea (Seoul, Republic of Korea), SYBR Green Realtime PCR Master Mix (QPK-201), purchased from Toyobo (Osaka, Japan), Chloroform (372978), 2-Propanol (109827), and Ethanol (51976) purchased from Sigma-Aldrich Co. (St. Louis, MO, USA) were employed. The following primer sequences were used: AQP3 (FW: 5′-ACG GTG GTT TCC TCA CCA TC-3′/RV: 5′-GGC TGT GCC TAT GAA CTG GT-3′), HAS2 (FW: 5′-ATT ACC CAG TCC TGG CTT CG-3′/RV: 5ʹ-CCT GTG GAA GAC TCA GCA GAA-3′), FLG (FW: 5′-TGA GGC ATA CCC AGA GGA CT-3′/RV: 5′-CTG TAT CGC GGT GAG AGG AT-3′), GAPDH (FW: 5′-GTC TCC TCT GAC TTC AAC AGC G-3′/RV: 5′-ACC ACC CTG TTG CTG TAG CCA A-3′). The antibodies against HAS3 (1:500 dilution, PA5-76964) and donkey anti-rabbit IgG Alexa 488 (1:2000 dilution, A21206) were purchased from Invitrogen. Immunofluorescence was analyzed using Image-It Fixative solution (FB002) purchased from Invitrogen, Triton X-100 (93443) from Sigma-Aldrich Co., Tween 20 (T1027) from Biosesang (Yongin, Korea), and Fluoroshield with DAPI (F6057) from Sigma-Aldrich Co.

### 3.2. Preparation of TMP

Initially, the dehydrated fruiting bodies of *T. mesenterica* were finely milled using a blender (HappyCall, Seoul, Republic of Korea). The powder was blended with distilled water (DW) at a ratio of 1:100 (*w*/*w*). Subsequently, enzymes were added to the mixture at specific concentrations relative to the volume of DW; Viscozyme L (0.25%), Pectinex XXL (0.25%), and Performase GSM 80 (0.5%). The mixture was subjected to shaking at 50 °C, 150 rpm for 2 h to facilitate enzymatic reactions. Subsequently, to inactivate the enzymes, the solution was incubated at 90 °C for 10 min in a water bath and then cooled sufficiently. The solution was subsequently centrifuged at 8000 rpm for 20 min, and the supernatant was harvested. Ethanol was then mixed to the supernatant to adjust the alcohol content to 70%, and the solution was left overnight to allow polysaccharide precipitation. The precipitated polysaccharides were collected and freeze dried to obtain white flake-like TMP powder, with a yield of 26.74% ± 2.94, which was calculated based on three replicates.

### 3.3. TPC Assay

To determine the TPC of TMP, the samples were diluted in DW to a concentration of 500 ppm. A standard curve was constructed using five different concentrations of glucose (0, 75, 150, 300, and 600 ppm). Approximately 100 μL of each sample was transferred into an Eppendorf tube, and 100 μL of 5% phenol solution was added afterward. The mixture was then vortexed thoroughly. Then, 500 μL of 98% sulfuric acid solution was transferred to the Eppendorf tube, and the mixture was vortexed again and placed at room temperature for 30 min. Then, 200 μL of the reaction mixture was aliquoted to a 96-well plate and the absorbance was detected at 485 nm with a microplate reader (UV/VIS spectrophotometer (Thermo Fisher Scientific, Waltham, MA, USA).

### 3.4. Structural Elucidation of TMP

#### 3.4.1. Determination of the MW of TMP

The MW of TMP was determined using HPSEC. The analysis was carried out on an Agilent 1260 Infinity LC system (Agilent Technologies, Santa Clara, CA, USA) installed with a Superdex 75 10/300 GL column (1.0 × 30 cm; GE Healthcare Life Sciences, Chicago, IL, USA) and a refractive index detector (RID). Each sample (10 mg/mL) was eluted with 50 mM ammonium formate buffer (pH 5.5) at a flow rate of 0.5 mL/min at ambient temperature. The MW was determined based on a calibration curve prepared using standard pullulans (P-5, 10, 20, 50, 100, and 800; Showa Denko Co. Ltd., Tokyo, Japan).

#### 3.4.2. Monosaccharide Composition Analysis of TMP

The monosaccharide composition of the TMP was evaluated according to the method of Dai et al. [19], with minor modifications. In brief, TMP (0.5 mg) was hydrolyzed in 2 M trifluoroacetic acid (TFA) at 121 °C for 90 min. The resulting hydrolysates were derivatized with 1-phenyl-3-methyl-5-pyrazolone (PMP) in 0.3 M sodium hydroxide (NaOH) at 70 °C for 100 min. The PMP-labeled derivatives were subsequently analyzed using UV-detected high-performance liquid chromatography (HPLC) on a Shimadzu system (Kyoto, Japan) equipped with an Acclaim 120 C18 column (Thermo Fisher Scientific). The detailed chromatographic conditions are summarized as follows: (1) eluent: 0.1 M sodium phosphate buffer (pH 6.7): acetonitrile (82:18), (2) temperature: 30 °C, (3) flow rate: 1 mL/min, and (4) injection volume: 10 μL.

#### 3.4.3. Methylastion Analysis of TMP

To determine the glycosidic linkages of TMP, the samples were converted into partially methylated alditol acetates (PMAAs) following the Hakomori method [20] with slight modifications. Briefly, 0.5 mg of polysaccharide was diluted in 1 mL of DMSO containing 20 μL of glycerol and subjected to permethylation with iodomethane and sodium methylsulfinyl carbanion. Methylated products were purified using a C18 Sep-Pak cartridge (Waters, Milford, MA, USA). The carboxyl groups of the uronic acids were subsequently reduced with lithium triethylborohydride in tetrahydrofuran, followed by neutralization with acetic acid, and the products were collected on a C18 Sep-Pak column. The reduced samples were then hydrolyzed in 1 M TFA at 121 °C for 90 min, reduced with sodium borohydride at 25 °C for 150 min, and acetylated with acetic anhydride at 100 °C for 150 min [21]. The resulting PMAAs were detected using gas chromatography–mass spectrometry (GC-MS, Agilent Technologies) equipped with a SP2380 capillary column (30 m × 0.25 mm, 0.2 μm film thickness) (Supelco, Bellefonte, PA, USA). The operating conditions for GC-MS are summarized as follows: (1) injection temperature: 250 °C, (2) detection temperature: 280 °C, (3) oven temperature: 60 °C (1 min) → (30 °C/min) → 150 °C → (1 °C/min) → 180 °C → (1.5 °C/min) → 231 °C → (30 °C/min) → 250 °C (10 min), and (4) carrier gas: N_2_ (1.0 mL/min).

### 3.5. Mechanistic Investigation of TMP in Skin Cell Moisturization

#### 3.5.1. Cell Culture

HaCaT cells, a human keratinocyte cell line, were incubated in Iscove’s Modified Dulbecco’s medium (IMDM) supplemented with 10% fetal bovine serum (FBS) and 1% antibiotic and antimycotic solutions. The cells were maintained in a humidified incubator at 37 °C with 5% CO_2_ for 24 h to ensure optimal growth conditions.

#### 3.5.2. Cytotoxicity

HaCaT cells were aliquoted into 96-well plates at a density of 5 × 10^4^ cells/mL (200 μL/well) and incubated at 37 °C, 5% CO_2_ for 24 h. After incubation, the cells were exposed to the samples (TMP, TFP, and HA) at various concentrations (2.5, 5, 10, 25, 50, 100, 250, 500, 1000 μg/mL) and further incubated under the same conditions for an additional 21 h. Each well received 20 μL of MTT solution at a concentration of 5 mg/mL and the cells were incubated for another 3 h under identical conditions. Following incubation, the cell culture medium was gently removed and 150 μL of DMSO was introduced into each well to solubilize formazan crystals. The plates were gently shaken at room temperature for 10 min on an orbital shaker. The absorbance was detected at 570 nm using a microplate reader (UV/VIS spectrophotometer, Thermo Fisher Scientific). The cell viability was measured using the following formula:

$$Cell\;viability\;\left(\%\right)\;=\;\left(\frac{Absorbance\;of\;treated\;group}{Absorbance\;of\;control\;grop}\right)\;\times \;100$$

#### 3.5.3. q-RT PCR

HaCaT cells were distributed on 6-well plates at a density of 3 × 10^5^ cells/mL (2 mL/well) and exposed at 37 °C in 5% CO_2_ for 24 h. Serum starvation was performed for 24 h, followed by sample treatment for another 24 h. Total RNA was collected using TRIzol reagent following the manufacturer’s instructions and kept at −70 °C until use. cDNA was synthesized from an equal concentration of RNA using the cDNA Synthesis kit. Real-time PCR was conducted using the SYBR Green Realtime PCR Master Mix on a QuantStudio5 system (QuantStudio5 Real Time PCR, Thermo Fisher Scientific). GAPDH was utilized as an endogenous control for normalization, and the target gene mRNA levels (AQP3, HAS2, and FLG) were expressed relative to those of the control group.

#### 3.5.4. ICC

In an 8-well chamber plate, HaCaT cells were seeded at a density of 8 × 10^4^ cells/mL (400 μL/well) and incubated at 37 °C in a 5% CO_2_ for 24 h. The cells were incubated in media without FBS for 24 h. The cells were exposed to the samples for 24 h under the same conditions and stabilized with a fixative solution for 15 min at room temperature. Cells were then permeabilized with 0.1% Triton X-100 and 0.01% Tween 20 in PBS for 20 min at room temperature. After blocking with 2% BSA solution, the cells were incubated with anti-HAS3 antibody at 4 °C for 24 h. After five washes with TBST, cells were incubated with secondary anti-rabbit IgG Alexa Fluor 488 and counterstained with DAPI. The cells were imaged using an Olympus IX83 microscope (Olympus, Tokyo, Japan) equipped with a LUCPLFLN 20× objective. Fluorescence images were captured under identical laser power settings to ensure consistency across samples. Quantification of fluorescence intensity was performed using the cellSens Dimension software 3.1. The line profile function of the software was used to measure fluorescence intensity along a defined region of interest (ROI) within the cells. To normalize the fluorescence intensity, GFP (HAS3) fluorescence intensity was divided by DAPI fluorescence intensity. The normalized HAS3 fluorescence intensity for each experimental group was calculated as follows:

$$HAS3\;fluorescence\;intensity\;\left(\%\right)\;=\;\left(\frac{Fluorescence\;intensity\;of\;treated\;group}{Fluorescence\;intensity\;of\;control\;group}\right)\;\times \;100$$

All measurements were performed under consistent image processing parameters to minimize variability. Data were analyzed and presented as normalized mean fluorescence intensity values.

#### 3.5.5. Statistical Analysis

All experiments were conducted in triplicate to ensure the reliability of the results, and data are presented as the mean ± standard deviation (SD). Statistical analysis was performed using Microsoft Excel (Microsoft Corporation, Redmond, WA, USA). One-way ANOVA was used to analyze differences between groups followed by post hoc Bonferronic test. Statistical significance was defined as a *p*-value less than 0.05.

## 4. Conclusions

*Tremella mesenterica* represents an edible natural resource, and TMP demonstrates cost-effectiveness due to its high yield (26.74 ± 2.94%) achieved through enzymatic extraction, contributing to sustainability and eco-friendliness. Structurally, TMP is composed of mannose (as the main backbone), xylose, glucuronic acid, and glucose, forming a highly branched and complex polysaccharide structure that may influence its biological effects, such as skin cell moisturization. In vitro studies have shown that TMP significantly upregulates key hydration-related factors in keratinocytes, including AQP3, HAS2, FLG, and HAS3, which are involved in skin hydration mechanisms.

However, further studies are required to validate its efficacy and safety. While the current findings are based on in vitro experiments, additional in vivo studies are essential to confirm TMP’s effectiveness and safety under physiological conditions. Despite these limitations, TMP’s unique structural properties and demonstrated moisturizing effects in vitro suggest that it could serve as a promising natural moisturizer with potential applications across diverse industries, including cosmetics, food, biopolymers, and pharmaceuticals.

## Figures and Tables

**Table 1:** Yield and total polysaccharide content of *Tremella* spp. with enzymatic treatment.

Origin of Polysaccharide	Type of Enzymes			Results	
	Viscozyme L	Pectinex XXL	Performase GSM 80	Yield (%)	* TPC (%)
*T. mesenterica*	-	-	-	6.21 ± 1.61	65.33 ± 5.39
	1	-	-	18.20 ± 3.79	86.91 ± 6.32
	-	1	-	15.48 ± 3.46	72.62 ± 7.48
	-	-	1	14.49 ± 3.34	83.52 ± 2.34
	1	1	1	16.63 ± 3.02	96.08 ± 3.08
	2	1	1	17.13 ± 3.73	69.29 ± 2.88
	1	2	1	20.64 ± 6.08	72.16 ± 2.07
	1	1	2	26.74 ± 2.94	98.82 ± 4.91
*T. fuciformis*	-	-	-	1.26 ± 0.21	50.46 ± 3.73
	1	1	2	2.28 ± 2.06	56.66 ± 1.70

* TPC: Total polysaccharide content, mean ± SD, *n* = 3.

**Table 2:** Chemical properties of TMP.

MW	143 kDa		
Component sugar	Mole (%)	Glycosidic linkage site	Ratio (%)
Mannose	59.8 ± 0.3	Terminal	11.8
		2-linked	0.9
		3-linked	19.6
		2,3-linked	30.1
		3,4-linked	2
		2,3,4-linked	0.9
Xylose	24.0 ± 1.2	Terminal	4.7
		2-linked	0.7
		3-linked	6.7
Glucose	5.2 ± 0.0	Terminal	1
		3-linked	0.5
		4-linked	0.6
		2,3-linked	4
Glucuronic acid	11.0 ± 0.8	Terminal	2.5
		2-linked	1.7
		3-linked	0.8
		4-linked	10.2
		2,4-linked	1.1

## Data Availability

The original contributions presented in this study are included in the article and Supplementary Material. Further inquiries can be directed to the corresponding author.

## Supplementary Materials

The following supporting information can be downloaded at: https://www.mdpi.com/article/10.3390/molecules31020278/s1, Figure S1: Elution pattern and MW-determination of polysaccharide from *T. mesenterica*.

## Abbreviations

The following abbreviations are used in this manuscript:

TMP	*T. mesenterica* polysaccharide
TFP	*T. fuciformis* polysaccharide
MW	Molecular weight
HA	Hyaluronic acid
AQP3	Aquaporin-3
HAS2	Hyaluronic acid synthase-2
HAS3	Hyaluronic acid synthase-3
FLG	Filaggrin
DW	Distilled water
ICC	Immunocytochemistry
