# Supplementary figures and images for: Structural Elucidation and Moisturizing Potential of a Polysaccharide Derived from Tremella mesenterica
Source: Molecules. 2026 Jan 13;31(2):278. doi: 10.3390/molecules31020278 (PMC12844443; doi:10.3390/molecules31020278)

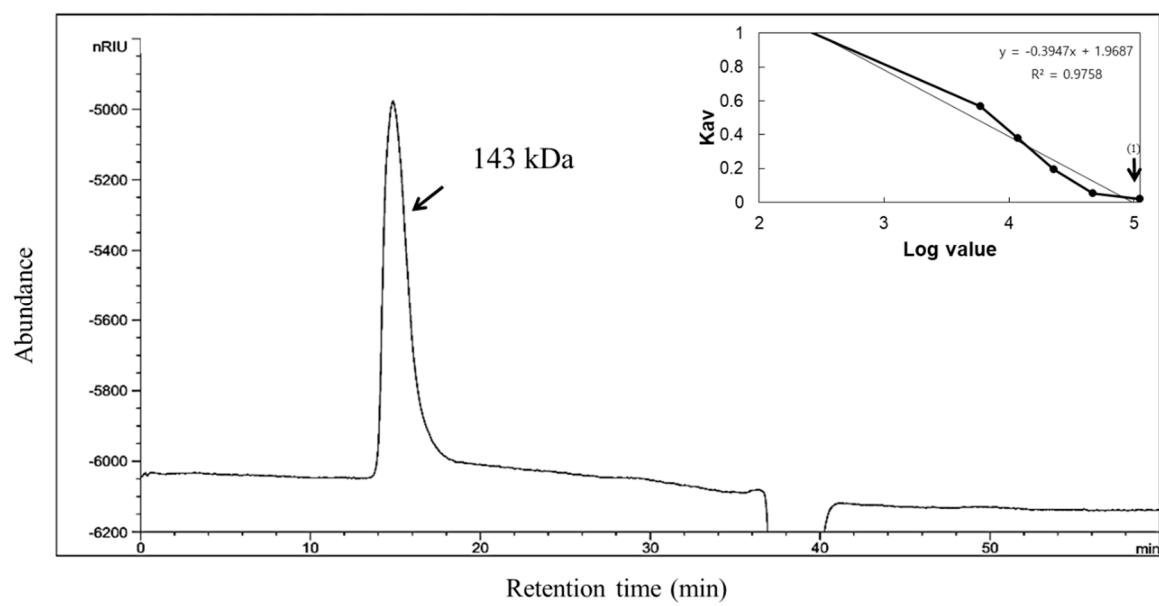

**Supplementary figure S1.** Elution pattern and MW-determination of TMP

Supplement: Supplementary file 1 [file molecules-31-00278-s001.zip › molecules-4022891-supplementary.pdf]
